# The relationship between live streamers’ self-disclosure and consumers’ purchase intention: The parallel mediating role of psychological distance and perceived homophily

**DOI:** 10.1371/journal.pone.0345061

**Published:** 2026-03-26

**Authors:** Yi Liu

**Affiliations:** School of Business and Management, Neusoft Institute Guangdong, Foshan, China; Dong-A University College of Business Administration, KOREA, REPUBLIC OF

## Abstract

As live streaming commerce continues to expand rapidly, the role of live streamers has emerged as a crucial factor. In an online environment with increasingly fierce competition and increasingly rich emotional needs of consumers, live streamers have gradually shown more self-disclosure to attract more consumers’ attention. While existing studies have indicated that the communication styles adopted by live streamers constitute a key factor influencing consumers’ purchasing behaviors, little is known about live streamers’ self-disclosure, a specific communication mechanism, is associated with consumers’ purchase intention, as well as the roles that psychological distance and perceived homophily between consumers and live streamers play in this process. Drawing on Social Penetration Theory, this study investigates the association between live streamers’ self-disclosure and consumers’ purchase intention, and analyzes the parallel mediating roles of psychological distance and perceived homophily in this relationship. The investigation employs regression analysis to examine 306 survey responses gathered from Chinese consumers. The results reveal that live streamers’ self-disclosure is positively associated with consumers’ purchase intention by diminishing the psychological distance between live streamers and consumers and enhancing perceived homophily; notably, psychological distance and perceived homophily exert significant parallel mediating effects in the association between live streamers’ self-disclosure and consumers’ purchase intention. Live streamers’ self-disclosure is linked to reduced audience psychological defensiveness via increased closeness, and to stronger identification through heightened similarity perception; both pathways are equally important in relation to higher purchase intention. This study contributes to a better understanding of how live streamers’ self-disclosure in live streaming contexts relates to consumers’ purchase intention, as well as the roles of psychological distance and perceived homophily between consumers and live streamers. It also provides a theoretical basis and practical implications for live streamers to engage in positive self-disclosure, to build closer connections and identification with consumers by reducing psychological distance and enhancing perceived homophily, and ultimately to support higher consumers’ purchase intention as well as the competitiveness of e-commerce platforms and enterprises.

## 1. Introduction

E-retailers are increasingly adopting digital marketing strategies that deliver more authentic information to consumers [1]. In recent years, e-commerce live streaming has sustained rapid growth as a novel format that integrates real-time social interaction with digital commerce [2]. Compared to traditional e-commerce, live streaming exhibits stronger presence, authenticity, and engagement [3], with live streamers serving as central figures [4, 5] whose behaviors are significantly associated with consumer perceptions, attitudes, and purchase intentions [6,7]. Beyond product demonstrations and instant Q&A [8,9], live streamers frequently engage in self-disclosure, which is defined as the act of sharing personal information with others [10], by narrating life stories and expressing emotional experiences in personalized ways [11].

This communicative practice aligns closely with Social Penetration Theory, which conceptualizes relationship development as a process driven by self-disclosure that progressively widens in topic range and deepens in personal intimacy [12]. In the live streaming context, even though interactions are often one-sided or parasocial, live streamers can still trigger psychological responses by revealing personal experiences, emotions, or values. Research shows individuals tend to develop stronger preferences for those who disclose more about themselves [12–14], and live streamers strategically leverage this tendency to attract and retain audience attention.

Although prior studies consistently report a strong association between live streamer communication styles and user purchase behavior [15–19], the underlying psychological micro-mechanisms remain largely unexplored, a persistent “black box” in the literature [9,20]. Much of the existing research aggregates live streamer behaviors into broad constructs such as “interactivity” or “credibility” and identifies mediators like immersion, parasocial relationships, or brand trust [9,15–18,20,21], yet rarely unpacks the sequential chain from “specific communication strategy → immediate psychological response → purchase decision”. In particular, how live streamers’ self-disclosure, through the lens of social penetration, is linked to viewers’ cognitive and affective responses and how these connect to purchase intention remains underexamined.

Notably, according to Social Penetration Theory, self-disclosure may be associated with consumer responses through two key psychological mechanisms. The first is psychological distance, referring to the subjective sense of closeness or separation between individuals [22,23]. When live streamers disclose personal experiences or emotions, they reduce interpersonal uncertainty, leading viewers to perceive a shorter social distance [23]. The synchronicity and unedited nature of live streaming further reinforce this perception, resulting in an association between reduced psychological distance and higher purchase intention [24–28].

The second mechanism is perceived homophily, defined as the extent to which viewers perceive similarity with the live streamer in values, lifestyles, or identity labels [12]. By strategically disclosing information (e.g., student status, frugal consumption habits, or hobbies), live streamers provide cues for social comparison. When viewers identify matching attributes, they infer “we are alike”, which enhances trust, reduces persuasion resistance, and is associated with higher purchase intention [29,30]. Features such as bullet comments and real-time responses in live streaming can continuously supply new similarity cues, reinforcing this psychological process.

To deepen understanding of communication dynamics in live streaming, this study focuses on live streamers’ self-disclosure as a specific behavior and examines its association with consumers’ purchase intention, with particular attention to the potential roles of psychological distance and perceived homophily. It aims to: (1) extend the boundary of self-disclosure research in the digital economy; (2) reveal the parallel mechanism of “closeness + similarity”; and (3) provide live streamers and platforms with a low-cost, high-conversion communication script that helps firms build sustainable competitive advantages.

## 2. Theoretical background and hypotheses development

Social Penetration Theory (SPT) posits that interpersonal relationships deepen through gradual and reciprocal self-disclosure [12]. As disclosures expand in breadth (range of topics) and depth (level of intimacy), interpersonal barriers are systematically reduced, accompanied by shifts in two key psychological perceptions: reduced psychological distance and enhanced perceived homophily [12,13]. Psychological distance refers to the subjective sense of closeness or separation between individuals [22,23], while perceived homophily denotes the extent to which individuals perceive similarity with others in values, lifestyles, or identity attributes [12,31]. According to SPT, sustained self-disclosure enables receivers to identify shared traits and reduce uncertainty, thereby shortening psychological distance and strengthening the cognition of “we are alike”, which lays the foundation for deeper relational bonds.

The live streaming commerce context offers a novel setting for applying SPT. Despite the absence of face-to-face interaction, live streaming is characterized by real-time interactivity, high engagement, and parasocial dynamics, all of which enable self-disclosure to efficiently trigger psychological penetration. First, real-time feedback mechanisms (e.g., bullet comments) allow live streamers to dynamically adjust the content and depth of their disclosures, accelerating the formation of closeness. Second, by sharing personal anecdotes, emotional experiences, or viewpoints, live streamers provide rich cues for social comparison, facilitating viewers’ identification of similarity. Third, within parasocial interactions, where audiences develop one-sided emotional bonds, viewers tend to perceive live streamers as “familiar others”, allowing even unidirectional disclosure to effectively reduce psychological distance and heighten perceived homophily [11,32]. Thus, SPT provides a robust theoretical lens for understanding how live streamers’ self-disclosure is associated with consumers’ psychological responses and behavioral intentions.

In live streaming commerce, self-disclosure serves not only an informational function but also constructs an authentic and relatable persona, mitigating the alienation typical of traditional commercial communication [33,34]. By sharing personal experiences, emotions, or opinions, live streamers signal sincerity and vulnerability, which is positively associated with higher perceived authenticity, credibility, and attractiveness [35,36]. These favorable impressions foster trust and positive attitudes toward both the live streamer and the promoted products [37,38], lower perceived risk, and are linked to higher purchase intention [33]. Thus:

H1: Live streamers’ self-disclosure is positively associated with consumers’ purchase intention.

Psychological distance refers to the subjective sense of separation between individuals [22,23]. According to Social Penetration Theory, self-disclosure is associated with reduced psychological distance by fostering emotional resonance and reducing uncertainty [12,23]. In live streaming, emotionally expressive or intimate disclosures act as “closeness cues”, leading viewers to perceive shorter social distance [25,26]. Real-time responses to bullet comments further reinforce the feeling of being attended to, thereby further narrowing psychological distance [24,39]. As psychological distance decreases, viewers are more likely to categorize the live streamer as an in-group member and transfer trust to recommended products, exhibiting higher purchase intention [27,28]. Therefore:

H2: Psychological distance mediates the association between live streamers’ self-disclosure and consumers’ purchase intention, such that greater self-disclosure is linked to reduced psychological distance, which in turn is associated with higher purchase intention.

Perceived homophily denotes the extent to which viewers perceive similarity with the live streamer in values, lifestyles, or identity labels in the live streaming context. Social Penetration Theory suggests that as self-disclosure increases in breadth and depth, receivers are more likely to identify shared attributes, strengthening similarity judgments [12]. Through strategic disclosures (e.g., revealing student status, frugal consumption habits, or hobbies), live streamers provide salient cues for social comparison [29]. When viewers recognize matching traits, they activate the cognition “we are alike”, which enhances trust via the similarity-attraction principle and fosters a sense of group belonging [30]. This identification effect reduces resistance to persuasion and is positively associated with willingness to support the live streamer through purchase behavior. Hence:

H3: Perceived homophily mediates the association between live streamers’ self-disclosure and consumers’ purchase intention, such that greater self-disclosure is linked to enhanced perceived homophily, which in turn is associated with higher purchase intention.

**Fig 1:**
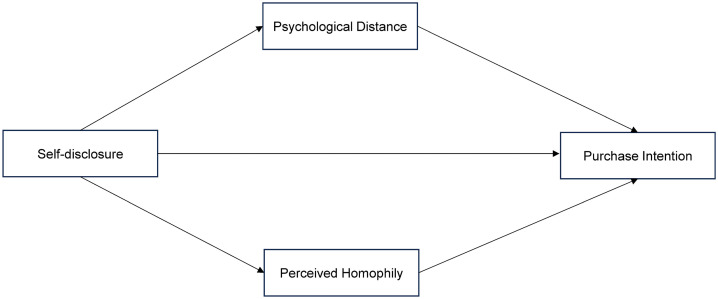
Conceptual framework in the research.

Together, H2 and H3 propose a parallel mediation model grounded in Social Penetration Theory, wherein self-disclosure is simultaneously associated with purchase intention through reduced psychological distance (a closeness pathway) and enhanced perceived homophily (a similarity pathway). The conceptual frameworkof this research showed by Fig 1.

## 3. Research methodology

### 3.1 Research design

This study employs a cross-sectional survey design to examine the relationship between live streamers’ self-disclosure and consumers’ purchase intention, as well as the parallel mediating effects of psychological distance and perceived homophily. Through an online questionnaire, consumers’ perceptions and evaluations of live streamers’ self-disclosure, consumer-live streamer psychological distance, and perceived homophily during live streaming were obtained; based on these survey data and analyses, the potential relationship between these factors and consumers’ purchase intention was explored.

The distributional characteristics of demographic variables sketch the basic profile of the sample and help readers assess its representativeness. At the same time, to enhance internal validity, this study treats demographic variables—gender, age, education, monthly disposable income and online shopping experience—as control variables. Previous research has shown that income level and online shopping experience significantly influence consumers’ purchase decisions in live streaming commerce context [34,40]. Therefore, these variables serve the dual purpose of describing sample characteristics and controlling statistical confounders in this study.

### 3.2 Samples

The sample size of this study was 306. According to the results of the sample analysis, regarding gender distribution, the sample comprises 130 male participants and 176 female participants, accounting for 42.48% and 57.52%. In terms of education, most of them are specialists and undergraduates, accounting for 79.40%. Regarding age distribution, the majority of participants are aged 18–25 and 26–35, comprising 70.59% of the total. Overall, the research subjects are relatively young and have a high acceptance of live-streaming commerce, which aligns with the research design requirements. The collected data samples are representative. The specific results of the descriptive statistical analysis are presented in (Table 1).

**Table 1:** Demographic characteristics.

Variable	Options	Frequency	Percent
Gender	Male	130	42.48
	Female	176	57.52
Education Level	High School and Below	31	10.10
	Associate Degree	60	19.60
	Bachelor’s Degree	183	59.80
	Graduate Degree and Above	32	10.50
Age	18-25	163	53.27
	26-35	53	17.32
	36-45	29	9.48
	46-55	24	7.84
	56-65	23	7.52
	Above 65	14	4.58
Monthly Disposable Income	3000 and Below	146	47.71
	3001-5000	54	17.65
	5001-8000	53	17.32
	8001-10000	25	8.17
	10001-20000	15	4.90
	20001 and Above	13	4.25
Online Shopping Experience	<1 year	32	10.46
	1-3 years	53	17.32
	3-5 years	109	35.62
	5-7 years	62	20.26
	>7 years	50	16.34

Note 1. Currency unit: Renminbi yuan (CNY).

Notes 2. Relative level (benchmarked against Guangdong’s monthly average of 4111 yuan in 2023): ≤ 3000 yuan ≈ 73% or below of Guangdong’s monthly average; 3001–5000 yuan ≈ 0.7–1.2 times of Guangdong’s monthly average; 5001–8000 yuan ≈ 1.2–1.9 times of Guangdong’s monthly average; 8001–10000 yuan ≈ 1.9–2.4 times of Guangdong’s monthly average; 10001–20000 yuan ≈ 2.4–4.9 times of Guangdong’s monthly average; > 20000 yuan ≈ more than 4.9 times of Guangdong’s monthly average.

### 3.3 Instrument development

All items in this research refer to the relevant literature and adopt mature scales with appropriate adjustments to the specific context of this research. To ensure the scale’s validity and scenario adaptability, I first invited three digital marketing scholars and a senior live streaming e-commerce operator to review the initial questionnaire. Revisions included refining item expressions (e.g., enhancing the pertinence to the live streaming scenario) and adjusting the order of items (placing demographic questions at the end). Then, a small-sample survey was conducted among 57 consumers with live streaming shopping experience. Analysis via SPSS 27.0 showed that the Cronbach’s α coefficient of each construct was greater than 0.7 (ranging from 0.729 to 0.923), indicating good internal consistency. Exploratory Factor Analysis (EFA) revealed a KMO value of 0.824, and the Bartlett’s Test of Sphericity was significant (p < 0.001). The factor loadings of items ranged from 0.653 to 0.842, with a cumulative variance explained rate of 69.23%, demonstrating acceptable construct validity. Based on these results, the expressions of 2 items were optimized to form the formal questionnaire. The items are in S1 File.

Live streamers’ self-disclosuremainly draws on the research of Hollenbaugh and Ferris [41], including three question items. One example item is “The live streamer shared a great deal of personal information and inner feelings”, where a high score indicates that the live streamer discloses more/wider/deeper self-information.

Psychological distance mainly draws on the studies of Edwards et al. and Xue [42,43], including 9 items. One example item of psychological distance is “The content presented by the live streamer makes me feel authentic and credible, enhancing my sense of closeness to him/her”. For the scale, a high score means that consumers perceive a closer psychological distance with the live streamer.

Perceived homophily mainly draws on Ladhari’s study [44], including 4 items. One example item of perceived homophily is “The live streamer’s ideas are similar to mine”. For the scale, a high score indicates that consumers perceive a higher degree of similarity with the live streamer.

Purchase itention mainly draws on Ki and Kim’s and Zheng’s studies [45,46], including 4 items. one example item of purchase intention is “I will consider purchasing the brand recommended by the live streamer If I need to buy this type of product.” For the scale, a high score represents a stronger purchase intention of consumers.

For the measurement of variables, the research employed 5-point Likert scale. The options of the scale numbered from 1 to 5, correspond to the respondent’s level of agreement: 5 indicates strongly agreement, while 1 signifies strongly disagreement.

### 3.4 Procedure and data collection

The study received approval from the Academic Research Committee of the School of Business Management at Neusoft Institute Guangdong. Prior to completing the online survey, all participants received detailed explanations about the study’s purpose and procedures. All participants in the study gave written informed consent, which ensured their full awareness of the research’s aims, methodologies, potential advantages, risks, and their own rights. During the entire course of the research, data were gathered on an anonymous basis, and the survey questionnaires contained no sensitive personal data. In the subsequent reporting of research findings, the relevant survey data were kept confidential in the strictest manner to protect the privacy of the respondents. The study qualifies for exemption from ethical review and has obtained a written exemption decision from the Academic Research Committee of the School of Business Management at Neusoft Institute Guangdong.

This study adopted snowball sampling, and questionnaires were distributed and data were collected through the online platform “SoJump”. Respondents were Chinese viewers who had live streaming viewing or consumption experiences on various e-commerce platforms in China. First, respondents who met the condition of “having watched e-commerce live streams in the past month” were invited to fill out the questionnaire through social channels such as WeChat (groups) and QQ (groups); after completing the questionnaire, participants could share the link with more live streaming audiences, and so on to achieve sample diffusion. Consumers who did not have such behaviour were excluded by setting “Have you watched or followed live streamers’ live streams online?” as a filter in the questionnaire. The first page of the questionnaire clearly stated: “Please recall the most recent live streaming that you watched completely and left an impression on you, and answer the following questions regarding the live streamer and products in that live streaming.” Geographical screening was automatically completed by SoJump’s built-in function “IP = Mainland China” to ensure that respondents were audiences in China. The questionnaires were distributed from March 19 to June 17, 2024. In this study, 343 questionnaires were initially distributed. Following the removal of invalid responses, a final sample of 306 valid questionnaires was obtained. Invalid questionnaires refer to those that are not suitable for analysis, such as those with incomplete answers and obvious contradictions between questions. Therefore, the validity rate of the questionnaires is 89.2%.To mitigate the potential interference of common method bias on the research findings, this study adopted targeted ex ante control measures during the questionnaire design and data collection phases: First, an anonymous response strategy was implemented. On the cover page of the questionnaire, respondents were explicitly informed that all data would be used exclusively for academic research purposes, and the information provided would be kept strictly confidential with no right or wrong answers. This was intended to guide respondents to answer independently based on their true perceptions, thereby reducing the risk of social desirability bias. Second, reverse scoring was applied to a subset of measurement items. For instance, positively worded items such as “The live streamer went into great depth when sharing personal information and inner feelings” were rephrased into reversed expressions like “This live streamer didn’t deeply share personal information and inner feelings”, which helped avoid systematic bias arising from respondents’ answering inertia.

### 3.5 Data analysis

Data analysis in this study was performed using SPSS 27.0 and PROCESS. Descriptive statistics for each variable and the correlation coefficients among variables were calculated via SPSS 27.0. PROCESS Model 4 was employed to test the parallel mediating effects, with gender, age, educational level, monthly disposable income, and online shopping experience set as covariates, which were incorporated into all regression paths of the mediating model. The Bootstrap method (with 5,000 resamples) was used to examine the significance of the mediating effects. All results of the path analysis were derived from the model after controlling for the aforementioned variables, so as to eliminate the interference of demographic factors on the relationships among the core variables.

For reliability and validity tests, Cronbach’s α coefficient was used to measure the internal consistency of items, with the threshold value set at ≥ 0.70 [47]. Composite reliability (CR) was employed to test construct reliability, with the threshold value set at ≥ 0.70 [48]. Average variance extracted (AVE) was applied to assess convergent validity, with the threshold value set at ≥ 0.50 [49]. For the path effect test, the indirect effect was determined to be significant if the 95% confidence interval constructed via the Bootstrap method did not contain zero [50].

## 4. Results

### 4.1 Assessment of measurement quality

Prior to hypothesis testing, this study evaluated the overall fit of the measurement model. The results indicated an acceptable-to-good model fit: χ²/df = 2.973, RMSEA = 0.080, CFI = 0.923, and TLI = 0.911.

Second, reliability and convergent validity were well supported. The CA, CR, and AVE for each variable as shown in (Table 2). As can be seen from the table, CA and CR were both greater than 0.8, AVE was both greater than 0.5. In addition, all factor loadings were above 0.6 (item-level factor loadings are presented in Supporting Information S1 File). These metrics meet or exceed conventional thresholds, confirming strong internal consistency and convergent validity.

**Table 2:** The results of the reliability analysis in the survey.

Variables	Cronbach’s α	AVE	CR
Self-disclose	0.832	0.627	0.834
Psychological Distance	0.938	0.630	0.939
Perceived homophily	0.816	0.526	0.815
Purchase Intention	0.866	0.624	0.869

Note: CA = Cronbach’s α; CR = Composite reliability, AVE = Average variance extracted.

Third, discriminant validity was established using the Fornell-Larcker criterion. As presented in (Table 3), the square root of the AVE for each construct was greater than its correlations with all other constructs, indicating that each latent variable captures a distinct underlying concept [49].

**Table 3:** Discriminant validity of measures used in measurement and correlation matrix.

	SD	Mean	1	2	3	4
Self-disclose	3.310	0.735	0.792			
Psychological Distance	2.574	0.777	−0.587^**^	0.794		
Perceived homophily	3.216	0.680	0.542^**^	−0.681^**^	0.725	
Purchase Intention	3.297	0.745	0.509^**^	−0.761^**^	0.679^**^	0.790

Note 1: ** denote significance levels of 0.01.

Note 2: The figures located along the diagonal represent the square root of the AVE; and the figures outside the diagonal represent the correlation coefficients between the various constructs.

Given that all data in this study were derived from respondents’ self-reports, Harman’s single-factor test was adopted for the post hoc assessment of common method bias [51]. All measurement items of latent variables were subjected to unrotated exploratory factor analysis (EFA). The results showed that a total of 4 common factors with eigenvalues greater than 1 were extracted, accounting for 65.35% of the cumulative explained variance; among these factors, the first common factor explained 32.52% of the variance, which is below the critical threshold of 40%. The aforementioned results indicated that there was no serious issue of common method bias in this study, and thus it would not exert a substantive impact on the core conclusions.

In summary, the measurement model demonstrated satisfactory reliability, convergent and discriminant validity, and minimal concern regarding common method bias, thereby providing a robust foundation for subsequent hypothesis testing.

### 4.2 Hypothesis testing

To test the impact of the independent variable live streamers’ self-disclosure on the outcome variable consumers’ purchase intention (H1), a direct effect analysis was first conducted. The findings indicated that the total effect of live streamers’ self-disclosure on consumers’ purchase intention was significant (β = 0.487, p < 0.001), yet its direct effect was not significant (β = 0.040, p = 0.377, 95% CI [−0.049, 0.130]). This indicated that the live streamers’ self-disclosure did not exert a significant direct influence on consumers’ purchase intention, and thus H1 was not supported. The findings are displayed in (Table 4).

**Table 4:** Results of the parallel mediation effect analysis.

Effect	Path	β	LLCI	ULCI	p
Total effect	SD- > PCI	0.487	0.389	0.585	p < 0.001***
Direct effect	SD- > PCI	0.040	−0.049	0.130	0.377
Indirect effect	SD- > PD- > PCI	0.296	0.210	0.387	p < 0.001***
Indirect effect	SD- > PH- > PCI	0.151	0.056	0.255	p < 0.001***
Indirect Effect Difference	PD-PH	0.146	−0.008	0.299	/

Note 1: SD: Self-disclosure; PCI: Purchase Intention; PD: Psychological Distance; PH: Perceived homophily.

Note 2: The model has controlled for the effects of gender, age, education level, monthly disposable income, and online shopping experience.

**Fig 2:**
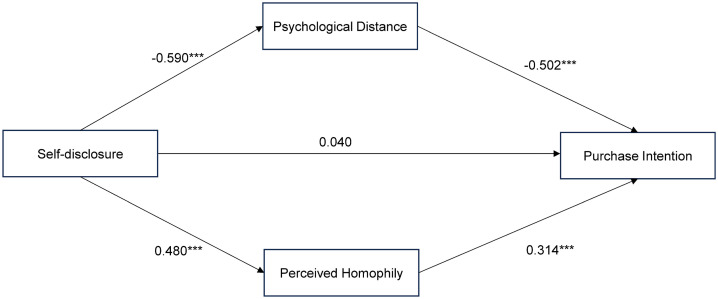
Path coefficient results of the parallel mediation model.

For the parallel mediation model (H2/H3) of live streamers’ self-disclosure → consumer-live streamer psychological distance/perceived homophily → consumers’ purchase intention, mediation effect tests were further performed. The results showed that live streamers’ self-disclosure exerted a significant negative effect on psychological distance (β = −0.590, p < 0.001) and a significant positive effect on perceived homophily (β = 0.480, p < 0.001). Meanwhile, psychological distance had a significant negative effect on consumers’ purchase intention (β = −0.502, p < 0.001), and perceived homophily had a significant positive effect on consumers’ purchase intention (β = 0.314, p < 0.001). Based on 5,000 Bootstrap resamples, the indirect effect coefficients of the two mediation paths were 0.296 (p < 0.001, 95% CI=[0.212, 0.380]) and 0.151 (p < 0.001, 95% CI=[0.098, 0.204]), respectively, with both CIs excluding zero. Therefore, both H2 and H3 were supported, indicating that the parallel mediating effects of psychological distance and perceived homophily in the path of “live streamers’ self-disclosure → consumers’ purchase intention” were significant. Moreover, as the direct effect of live streamers’ self-disclosure on consumers’purchase intention was not significant (β = 0.040, p = 0.377), this indicated that psychological distance and perceived homophily jointly exerted a full mediating role in the relationship between self-disclosure and purchase intention. The path coefficient results of the model are presented in Fig 2.

Notably, although the parallel mediating effects of social distance and perceived homophily are both significant, the difference in their effect sizes is not statistically significant (Δβ = 0.146, CI [−0.008, 0.299]). Coupled with the fact that the two constructs are highly negatively correlated (r = −0.681, p < 0.001), this suggests that they may not be entirely independent but instead jointly reflect a higher-order construct.

## 5. Discussion

### 5.1 Discussion of results

This study sought to explore the relationship between live streamers’ self-disclosure and consumers’ purchase intention in the context of e-commerce live streaming, with a specific focus on the mediating roles of psychological distance and perceived homophily. The results indicate that self-disclosure is not directly associated with purchase intention (H1 is not supported). However, the association between self-disclosure and purchase intention is accounted for by two indirect pathways: one through reduced psychological distance between consumers and live streamers (H2 supported), and the other through heightened perceived homophily (H3 supported). This pattern suggests that the link between self-disclosure and consumer decision-making is not driven by a direct effect of the behavior itself, but is embedded within the parallel psychological processes of perceived closeness and similarity that it evokes. This result is consistent with the core proposition of the Social Penetration Theory: by taking the initiative to disclose personal experiences, emotions, and opinions to audiences, live streamers gradually reduce the audiences’ perceived uncertainty toward them, thereby fostering deeper intimate relationships during real-time interactions [33,34]. In the context of live streaming commerce, such penetrative communication transforms live streamers from mere information providers into members of the audiences’ “in-group” who are worthy of trust and identification, which in turn enhances consumers’ purchase intention. In comparison with existing studies [15–18,21], this paper further verifies that self-disclosure, as a specific communication mechanism, can exert a notable persuasive effect in the highly real-time and highly visual field of live streaming commerce. However, this relationship is contingent upon the reduction of psychological distance and the enhancement of perceived homophily.

One hand, psychological distance between live streamers and consumers plays a mediating role in the in the relationship between the live streamers’ self-disclosure and the consumers’ purchase intention. When audiences perceive a high level of similarity with live streamers, they tend to feel “closer” to the live streamers, are more likely to regard the live streamers as peers rather than mere salespersons, which leads to reduced defensive mechanisms and enhanced information acceptance. The mediating effect of psychological distance in this study echoes a previously identified “trust → purchase intention” pathway in Chinese live streaming contexts [33], confirming that “shortening distance” is equally crucial in collectivistic cultures.

On the other hand, perceived homophily between live streamers and consumers also plays a mediating role in the in the relationship between the live streamers’ self-disclosure and the consumers’ purchase intention. Existing studies have shown that similarity in values/lifestyles can enhance source persuasiveness [16]. The mediating effect of perceived homophily in this study echoes the assertion by Deng et al. that similarity is associated with increased consumer purchase intention [52]. However, unlike existing research that emphasizes the moderating role of perceived similarity [53], this study argues that the similarity between live streamers and audiences plays a significant mediating role in the impact of live streamers’ self-disclosure on audience trust. It can transform “similarity identification” into immediate purchase motivation rather than merely remaining at the level of situational moderation, thereby providing a quantifiable psychological pathway for the design of live streamers’ differentiated personas.

Both H2 and H3 are supported, indicating that psychological distance and perceived homophily form a parallel mediating pathway. Social Penetration Theory posits that relationship deepening relies on two interchangeable resources: “sense of closeness” and “sense of similarity” [32,54]. The findings of this study reveal no significant difference in the magnitude of the two indirect effects, suggesting that live streamers’ self-disclosure not only reduces audience defensiveness by shortening social distance but also enhances identification by activating the inference of “we are similar”—with both pathways being equally important. In other words, while perceiving live streamers as “closer”, audiences also view them as “more like me”, and the superposition of this parallel identification drives higher purchase conversion. Therefore, when implementing self-disclosure strategies in live streaming commerce, equal emphasis should be placed on “shortening distance” and “highlighting similarity” to maximize the effect of interpersonal penetration. This study is the first to simultaneously verify the equal-weight effect of the “closeness + similarity” parallel pathways in live streaming commerce, supplementing Social Penetration Theory with evidence of a parallel mechanism in “real-time, highly visual” contexts.

Furthermore, the high correlation between consumer-live streamer psychological distance and perceived homophily, together with the results of the mediating effect comparison, also indicate that in live streaming scenarios, “psychological distance” and “perceived homophily” between live streamers and consumers may not exist in isolation but instead jointly reflect a higher-order construct (e.g., “interpersonal connection”). This finding merits further attention in future research.

### 5.2 Theoretical contributions

Grounded in Social Penetration Theory (SPT), this study isolates self-disclosure as a distinct interpersonal behavior from broader constructs commonly used in live streaming research, such as general interactivity, perceived trustworthiness, or parasocial engagement. Although prior work has established that live streamer communication influences consumer purchase intention [15–19], self-disclosure is often treated as a background cue or an undifferentiated element of authenticity, leaving its psychological mechanisms unexamined. This study addresses this gap by demonstrating that the association between live streamers’ self-disclosure and consumers’ purchase intention operates exclusively through two parallel mediating pathways: reduced psychological distance and enhanced perceived homophily. This finding advances theory in three key ways.

First, it extends the applicability of SPT into high visibility, real time digital commerce environments. While SPT posits that relational intimacy emerges from the joint development of closeness through reduced distance and similarity through homophily [12], the relative roles of these processes in mediated, quasi social interactions have remained empirically untested. The results of this study show that both mediators exhibit statistically equivalent effect sizes and jointly account for the total effect, indicating that in live streaming contexts, closing interpersonal gaps and highlighting shared attributes are equally important. This not only confirms SPT’s relevance in algorithmically mediated settings but also operationalizes its core principles into measurable psychological constructs.

Second, the findings challenge the common assumption that psychological distance and perceived homophily function as independent mechanisms. The data reveal a strong negative correlation between the two mediators, with no significant difference in their indirect effects. This suggests they may represent complementary facets of a higher order construct, namely interpersonal bonding, rather than separate processes. From the perspective of self-disclosure, sharing personal information simultaneously reduces perceived separation and activates social categorization based on shared identity. In other words, reduced distance lowers cognitive and affective barriers, while perceived homophily strengthens identification and affiliation. Together, they serve a unified function in building relational connection. This integrative view moves beyond single mediator models and offers a more nuanced understanding of how emotional and cognitive processes co evolve in digital parasocial relationships.

Third, the study provides theory driven guidance for platform design and live streamer communication strategies. Current practices often emphasize either emotional warmth or demographic alignment in isolation. The results support a dual path approach that combines distance reduction and similarity enhancement. For example, a live streamer who shares a personal challenge while explicitly referencing a shared audience identity, such as student status or lifestyle values, can activate both psychological pathways simultaneously. Such mechanism informed communication is more theoretically grounded and potentially more effective than intuition based engagement tactics.

In summary, the contribution of this research lies not in reconfirming that live streamers influence purchase decisions, a well established consensus, but in precisely identifying how self-disclosure, as a theoretically distinct behavior, translates into consumer outcomes through two co equal psychological routes. By successfully applying SPT from face to face dyads to the dynamic, visual, and asymmetric context of live commerce, and by revealing its parallel pathway logic, this study opens new theoretical avenues for research on interpersonal influence in digital marketplaces.

### 5.3 Practical implications

This study finds that live streamers’ self-disclosure has no significant direct effect on consumers’ purchase intention; its influence operates entirely through the parallel mediating roles of psychological distance and perceived homophily. Therefore, merely increasing self-disclosure behaviors alone will not enhance purchase intention; what matters is whether the disclosed content effectively reduces psychological distance or strengthens perceived homophily. This insight carries critical implications for LSC practice.

For live streamers, first, it is essential to avoid equating “talking more” or “sharing personal anecdotes” with effective strategy. Instead, during the early stage of a broadcast or gaps between product switches, live streamers should deliberately share personal stories that align closely with their target audience’s identity, needs, or values (e.g., “I rely on this coffee even when staying up late continuously to edit videos”). Such disclosures simultaneously convey lived experience and group identity cues, thereby reducing the psychological distance with audiences and enhance the “sense of closeness”. Secondly, during the process of self-disclosure, live streamers can pay attention to the specific needs of consumers, especially emotional needs [55]. For instance, they can collect viewers’ interest tags in advance through bullet comments and communities, and embed “peer signals” in the disclosed content (e.g., being a student group or valuing cost-effectiveness similarly), enabling audiences to form the perception that “we are the same kind of people” and enhancing the persuasive effect. Targetedly allowing consumers to intuitively understand the characteristics and usage methods of products during live streaming [56] can significantly reduce consumers’ information search costs [57]. Meanwhile, it is suggested that live streamers employ emotional expression, such as sharing touching stories or personal experiences related to the products, to resonate with consumers using sincere language. In particular, live streamers are advised to focus on establishing unique and differentiated individual symbols or tags; the application of such emotional symbols can stimulate emotional resonance among audiences [58], thereby strengthening the perception of homophily between consumers and live streamers and improving their purchase intention for the products recommended by live streamers

From the standpoint of e-commerce platforms and enterprises, first, it is recommended that platforms and enterprises consider the matching degree between live streamer types and products in the process of selecting live streamers [59,60], ensuring that the live streamers’ persona naturally facilitates the activation of reduced psychological distance or enhanced homophily through self-disclosure, thereby indirectly boosting purchase intention. Second, it is suggested that e-commerce platforms or enterprises provide necessary training for live streamers [8,61], guiding them to engage in more effective self-disclosure,thereby influencing consumers’ internal perceptions, shortening psychological distance, and strengthening the cognition of homophily. Live streamers can be encouraged to present their authentic selves and emotions [62] to bridge the gap with the audience. By engaging in genuine and transparent self-presentation [63], trust can be established with the audience, which in turn facilitates positive and appropriate self-disclosure. For example, a “real story library” module can be added to live streamer training, requiring each live streamer to prepare 3–5 personal experiences related to the product category, and evaluating the frequency and growth rate of their disclosure in quarterly assessments. Finally, platforms and enterprises can also leverage digital resources and tools such as big data to provide e-commerce live streamers with precise user profiles and differentiated products [64,65]. By considering the type and characteristics of different products, platforms can assist live streamers in flexibly adjusting their levels of self-disclosure. This will help live streamers to effectively connect with consumers through self-disclosure, shorten the psychological distance between live streamers and consumers, and enhance consumers’ perception of similarity with live streamers, thereby promoting precise marketing for e-commerce platforms and enterprises, as well as enhancing purchase intention of consumers and competitiveness of the enterprises.

### 5.4 Limitations and future research

Although this study has made certain contributions, it is undeniable that there are also some limitations, and further research is needed in the future. First, this study adopts a cross-sectional design and relies on participants’ retrospective reports of their most recent live streaming viewing experience, which may affect the accuracy of the research conclusions. Capturing only self-disclosure, psychological distance, perceived homophily, and purchase intention at a single time point limits the inference of causal relationships; retrospective reporting of a single live streaming event may lead to “memory salience bias”—participants are more likely to retrieve highly salient instances that led to purchases, while omitting ordinary viewing experiences without purchases, which may exaggerate the “live streaming-purchase” relationship. Future research can combine real-time recording or platform backend behavioral data to reduce systematic biases caused by retrospective sampling; at the same time, adopt longitudinal or experimental designs to collect multi-timepoint data before live streaming, immediately after live streaming, and one week later, so as to provide more solid evidence for the causal chain of Social Penetration Theory in the context of live streaming commerce.

Second, the sample of this study is limited to eastern coastal cities in China, where there is high cultural homogeneity. Moreover, due to the anonymous questionnaire not collecting explicit demographic characteristics such as live streamers’ gender and ethnicity, it may not only compress the actual performance of the effects of psychological distance and homophily but also miss the amplifying or inhibiting effect of “explicit similarity” on perceived homophily. From the perspective of cultural traits, this sample group generally exhibits a high tendency toward collectivism and pays more attention to “group-level similarity” [66], which may make the weight of the homophily influence pathway higher than that in individualistic cultural contexts; at the same time, their indulgence tendency is relatively low, and their emphasis on material possession is also weak [67]. It is worth noting that in cross-gender and multi-ethnic live streaming scenarios, explicit characteristics such as gender, ethnicity, and language may moderate the effect of self-disclosure by shaping the formation of parasocial relationships between viewers and live streamers. Prior research has shown that when audiences perceive demographic similarity (e.g., in gender or ethnicity) with media figures, they are more likely to develop stronger parasocial bonds, which in turn heighten their receptivity to self-disclosing behaviors [68]. Consequently, such explicit similarity may amplify the pathways through which self-disclosure fosters perceived closeness and homophily in live commerce contexts. Based on this, future research can combine Hofstede’s cultural dimensions [67] to carry out cross-cultural comparative experiments, or design control scenarios of “consistent live streamer-audience gender/ethnicity” and “inconsistent live streamer-audience gender/ethnicity”, so as to test the differences in the influence pathway of “psychological distance/perceived homophily → purchase intention” between high-indulgence cultures (e.g., the United States) and low-indulgence cultures (e.g., China). This will further clarify the cultural applicable boundary of the model and provide theoretical support and practical reference for formulating differentiated communication strategies in the global live streaming commerce field.

Third, the current study only measured one-way self-disclosure using three items: “quantity-breadth-depth”, which did not cover multi-dimensional levels such as emotion (emotional sharing), behavior (behind-the-scenes highlights), and cognition (value statements), and also ignored two-way interactions such as bullet comments and voice calls. In the future, a multi-dimensional scale including emotion-behavior-cognition can be developed, or natural language processing can be used to real-time analyze bullet comments and voice to test which type of self-disclosure plays a greater role in shortening psychological distance and improving perceived homophily; experiments on “real-time Q&A-voice call interaction” can also be designed to compare the effects of one-way and two-way communication, so as to expand the framework of this study and provide refined scripts for live streamers. Similarly, individually perceived similarity can be reflected in multiple aspects such as values, attitudes, personality, hobbies, appearance, socioeconomic status, and ethnicity [69]. However, the measurement of perceived homophily in this study failed to fully cover these aspects, and thus may only partially reflect the effects generated by perceived homophily. Future research can expand the questionnaire measurement dimensions of perceived homophily to conduct a more comprehensive investigation.

In addition, this study did not collect the unit price of products in the questionnaire, which may have omitted the significant impact of price on purchase intention. For example, the difference in persuasion difficulty between products priced at 5 US dollars and 200 US dollars is sufficient to explain additional variance. In the future, price items should be directly included in questionnaire or experimental designs to systematically examine whether and to what extent price moderates the intensity of the effect of self-disclosure on purchase intention. Furthermore, it is worth noting that this study did not explore the factor of the relevance between self-disclosure and products, that is, whether the personal experiences shared by live streamers are truly related to the promoted products/services. Future research can set up control groups to test whether the relevance of live streamers’ self-disclosure to products (e.g., high relevance/low relevance/no relevance) exerts differentiated effects on shortening psychological distance and improving perceived homophily.

Finally, this study focuses on the relationship between live streamers’ self-disclosure and consumers’ purchase intention. It should be noted that purchase intention is not equivalent to actual purchase [70,71]. Therefore, future research can combine platform backend transaction data or field experiments to track consumers’ real purchase behaviors and test the relationship between live streamers’ self-disclosure and actual purchases; it can also record indicators such as payment amount and return rate simultaneously to comprehensively evaluate the long-term marketing effects of self-disclosure strategies.

## 6. Conclusion

The study investigates the relationship between live streamers’ self-disclosure and consumers’ purchase intention, with a particular focus on the parallel mediating roles of psychological distance and perceived homophily. The findings reveal that live streamers’ self-disclosure significantly relates to consumers’ purchase intention by reducing psychological distance and increasing perceived homophily. Both psychological distance and perceived homophily play significant mediating roles in the relationship between live streamers’ self-disclosure and consumers’ purchase intention. This suggests that reducing distance and enhancing similarity are equally important for boosting purchase intention, while their high correlation suggests that the two mechanisms can be viewed as complementary facets of building interpersonal connection in live-commerce settings.

This study enriches the extant literature by elucidating the underlying processes and relational dynamics between live streamers’ self-disclosure and consumer actions. By integrating social penetration theory with the constructs of psychological distance and perceived homophily, the research provides a comprehensive framework that explains how self-disclosure relates to consumers’ perceptions and intentions. This framework can guide future research on the role of self-disclosure in various contexts of digital marketing. The empirical results provide practical guidance for live streamers and digital platform managers to optimize engagement strategies.

## Supporting information

S1 File Data. (XLSX)
